# Development and validation of a novel model to predict pulmonary embolism in cardiology suspected patients: A 10-year retrospective analysis

**DOI:** 10.1515/med-2024-0924

**Published:** 2024-03-08

**Authors:** Fang Ling, Qiang Jianling, Wang Maofeng

**Affiliations:** Department of Cardiology, Affiliated Dongyang Hospital, Wenzhou Medical University, Dongyang, 322100, Zhejiang, China; Department of Biomedical Sciences Laboratory, Affiliated Dongyang Hospital, Wenzhou Medical University, Dongyang, 322100, Zhejiang, China

**Keywords:** prediction model, clinical symptoms, biomarkers, nomogram

## Abstract

As there are no predictive models for pulmonary embolism (PE) in patients with suspected PE at cardiology department. This study developed a predictive model for the probability of PE development in these patients. This retrospective analysis evaluated data from 995 patients with suspected PE at the cardiology department from January 2012 to December 2021. Patients were randomly divided into the training and validation cohorts (7:3 ratio). Using least absolute shrinkage and selection operator regression, optimal predictive features were selected, and the model was established using multivariate logistic regression. The features used in the final model included clinical and laboratory factors. A nomogram was developed, and its performance was assessed and validated by discrimination, calibration, and clinical utility. Our predictive model showed that six PE-associated variables (age, pulse, systolic pressure, syncope, D-dimer, and coronary heart disease). The area under the curve – receiver operating characteristic curves of the model were 0.721 and 0.709 (95% confidence interval: 0.676–0.766 and 0.633–0.784), respectively, in both cohorts. We also found good consistency between the predictions and real observations in both cohorts. In decision curve analysis, the numerical model had a good net clinical benefit. This novel model can predict the probability of PE development in patients with suspected PE at cardiology department.

## 1 Introduction

Pulmonary embolism (PE) is a serious and potentially life-threatening medical condition resulting from blood clot formation in one or more arteries in the lungs [1]. Patients with cardiovascular diseases and cancer [2], particularly those with heart failure, atrial fibrillation, and coronary artery disease, are at an increased risk of developing PE because of the pro-thrombotic state of their conditions [3]. Furthermore, patients who undergo cardiac surgeries or interventions [4], such as coronary artery bypass grafting and percutaneous coronary intervention, are at a higher risk of developing PE. PE can have a significant effect on the prognosis and quality of life of patients with cardiovascular diseases [5]; therefore, timely diagnosis and treatment are essential for improving patients’ outcomes [6]. Current diagnostic methods have limitations and may lead to unnecessary testing and delays in treatment [7].

Computed tomography pulmonary angiography (CTPA) is the gold standard diagnostic method for PE [8], but it is associated with high radiation exposure, contrast-induced nephropathy, and allergic reactions. Furthermore, CTPA may not be appropriate for some patients such as pregnant women or those with kidney disease. Therefore, there is a need for noninvasive and accurate methods to identify patients with high risk of PE. Several studies have been conducted to develop and validate models for predicting the risk of PE in various populations including emergency department patients [9], pregnant and postpartum women [10], and patients with underlying medical conditions such as cancer [11] and heart failure [12]. One example of a scoring system is the Wells score, which is widely used to assess the probability of PE in patients with suspected PE [13]. The Wells score is based on clinical variables such as prior deep vein thrombosis symptoms, clinical symptoms, and presence of limb edema. Other scoring systems [14] such as the Geneva score and the PE rule-out criteria have also been developed and validated in patients presenting with a primary complaint of shortness of breath or chest pain, and it is reasonable to use it for either of these symptoms.

In recent years, there has been increasing interest in developing predictive models for PE in specific populations. For example, Jen et al. developed a new model that outperforms existing predictive tools in all patients with PE [15]. Lin et al. developed a new clinical predictive model that can identify patients who are at high risk of venous thromboembolism and help provide medical intervention in patients with diabetes and the general population [16]. While PE predictive models have been developed and validated in various populations [17], there are still some shortcomings and deficiencies that need to be addressed. One limitation of existing PE predictive models is their lack of generalizability across different patient populations. For example, a model developed in a population of emergency department patients may not be applicable to patients in a primary care setting or those with underlying medical conditions such as cancer or heart failure. In addition, some PE predictive models may not fully capture the complex interactions between various risk factors and their contribution to PE development. For example, the Wells score, although widely used, does not include variables such as the presence of a hypercoagulable state, which may increase the risk of thromboembolism [18].

Currently, there is no widely accepted model or guideline for predicting the probability of PE in patients with cardiovascular disease. Therefore, there is a critical need for developing an accurate and reliable predictive model for PE in patients with cardiovascular diseases.

In this study, we bridge this gap by developing and validating a novel numerical model to predict the probability of PE in patients with cardiovascular disease. This model simplifies risk assessment and provides a user-friendly interface for medical practitioners to assess a patient’s risk level.

## 2 Methods

### 2.1 Patient enrollment and data collection

This retrospective study enrolled patients with suspected PE at the Department of Cardiology at the Affiliated Dongyang Hospital of Wenzhou Medical University from January 2012 to December 2021. The data of 995 subjects were collected from the hospital’s clinical research data platform, after baseline data clearing and extraction. The patients were randomly divided into a training cohort and a validation cohort at a ratio of 7:3.


**Ethical approval:** This study was approved by the Medical Ethics Committee of the Affiliated Dongyang Hospital of Wenzhou Medical University (No.: 2022-YX-160). The requirement for informed consent was waived. Patient records or information were anonymized and de-identified before our analysis. Our research was conducted in adherence with the Declaration of Helsinki.

### 2.2 Diagnostic criteria

**Figure 1:**
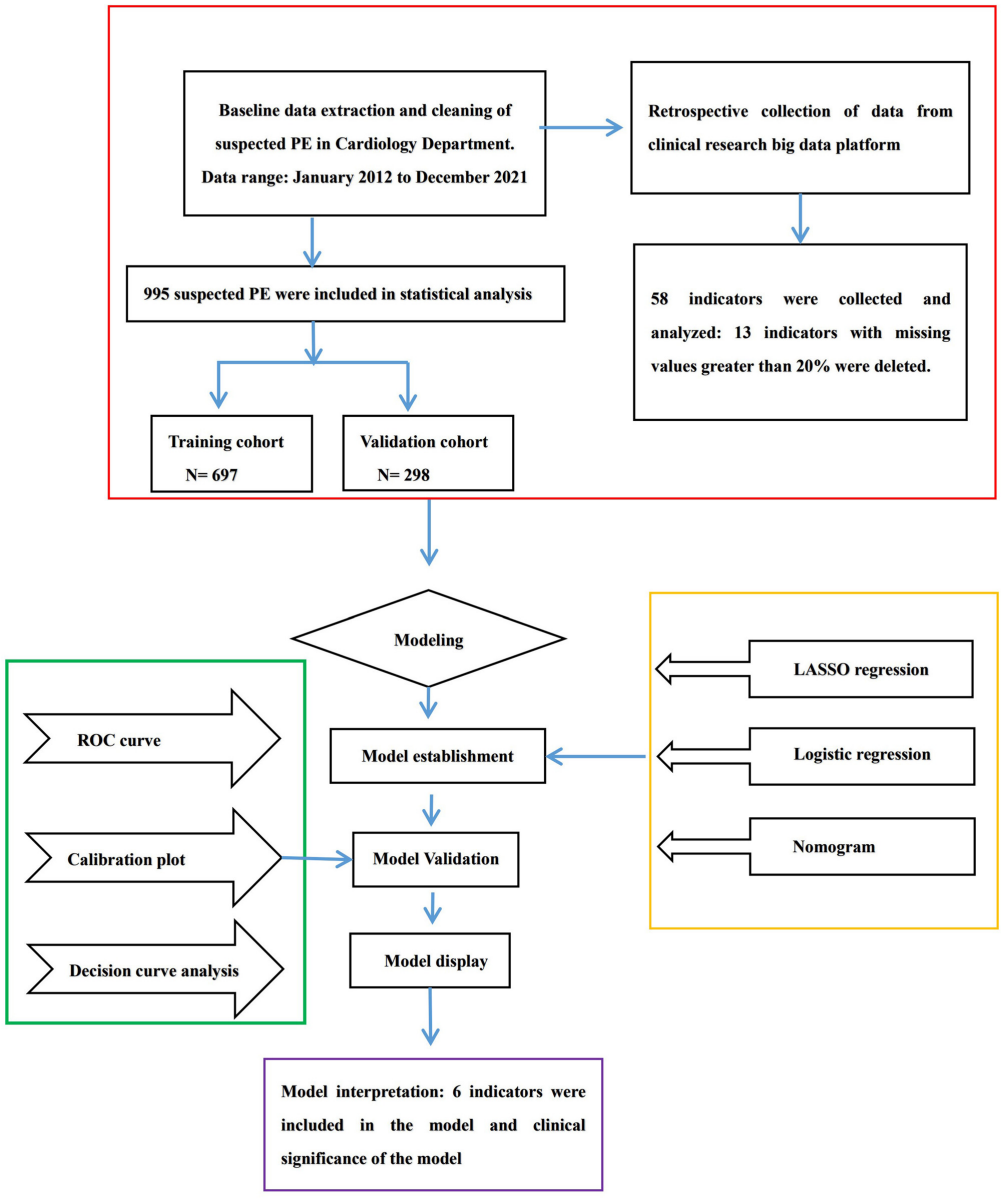
Flowchart of the steps for predicting PE diagnosis.

The diagnosis of PE in our study was based on the criteria outlined in the European Society of Cardiology Guidelines [19], and patients who had undergone CTPA examination were classified as those having suspected PE. The diagnosis was based on the presence of a filling defect in the pulmonary artery system, including the subsegment pulmonary artery, as seen on CTPA. In addition to CTPA results, we collected patients’ past medical history, clinical features, complications, and biomarker data using strictly defined indicators. For instance, we selected the lowest value of blood oxygen saturation, systolic blood pressure, and diastolic pressure from admission to CTPA, while the highest value was chosen for other indicators. A flowchart of the steps involved in PE prediction model is presented in Figure 1.

### 2.3 Statistical analysis

The data were analyzed using R Studio software for Windows. Categorical variables were presented as frequencies with percentages and were compared using either the *χ*² test or Fisher’s exact test. Continuous variables were expressed as mean values with standard deviations or medians with interquartile ranges and were compared using either Student’s *t*-test or Mann–Whitney *U* test. A total of 58 variables were collected for each subject. To ensure data reliability, 13 indicators with missing information in greater than 20% of patients were excluded. Multiple imputation techniques [20] using the “mice” package in R software were applied to impute the remaining missing predictor values. The optimal predictive features were selected using the least absolute shrinkage and selection operator (LASSO) regression analysis [21] with the “glmnet” package, and the numerical model was established using multivariate logistic regression analysis with the “rms” package. A nomogram was constructed using the “regplot” package in R software. The features were presented as odds ratios (ORs) with 95% confidence intervals (CIs). A two-sided *p*-value of less than 0.05 was considered statistically significant.

### 2.4 Model development, validation, and evaluation

In the training cohort, we employed LASSO regression to select the optimal predictive features and developed a multivariable logistic regression model to predict the probability of PE. To evaluate the performance of the model, discrimination, calibration, and clinical utility were assessed and validated in both cohorts. Discrimination was evaluated using the area under the receiver operating characteristic (ROC) curve (AUC) with the “pROC” package. Calibration was assessed with calibration curve analysis using the “calibrate” package. We performed decision curve analysis (DCA), clinical impact curve, and net reduction curve with the “rmda” package to quantify the net benefit under different threshold probabilities and determined the clinical utility of the model.

## 3 Results

### 3.1 Study population characteristics

In this study, we excluded 13 variables with missing information in more than 20% of patients, leaving 45 variables with missing data in less than 20% of patients (as shown in Appendix 1). The multiple imputation technique was used to impute the missing data for these 45 variables, which ranged from 0.00 to 12.46%. A total of 995 subjects with suspected PE were included, and the incidence of PE in our study was 17.98%. The baseline characteristics of patients with suspected PE at the cardiology department are presented in Table 1. We randomly divided the patients into the training cohort (*n* = 697) and the validation cohort (*n* = 298). The baseline characteristics of patients in the two cohorts are shown in Table 2. There was no significant difference in each indicator between the two cohorts, except for two indicators (platelet distribution width and thrombin time).

**Table 1:** Baseline characteristics of the study subjects

Variables	Total (*N* = 995)	No PE (*N* = 816)	PE (*N* = 179)	*p*
Sex, *n* (%)				0.150
Female	499 (50.2%)	400 (49.0%)	99 (55.3%)	
Male	496 (49.8%)	416 (51.0%)	80 (44.7%)	
Age (years)	76.0 [67.0; 82.0]	75.0 [65.0; 81.2]	78.0 [71.0; 84.0]	<0.001
Breathing (breaths/min)	22.0 [20.0; 26.0]	22.0 [20.0; 26.0]	24.0 [22.0; 28.0]	<0.001
Pulse (beats/min)	103 [89.0; 124]	102 [88.0; 120]	112 [98.0; 140]	<0.001
Systolic pressure (mmHg)	101 [91.0; 113]	102 [92.0; 115]	95.0 [87.5; 105]	<0.001
Diastolic pressure (mmHg)	54.0 [47.0; 63.0]	55.0 [48.0; 64.0]	51.0 [45.0; 57.5]	<0.001
Headache, *n* (%)				0.204
No	968 (97.3%)	791 (96.9%)	177 (98.9%)	
Yes	27 (2.71%)	25 (3.06%)	2 (1.12%)	
Dizzy, *n* (%)				0.474
No	957 (96.2%)	787 (96.4%)	170 (95.0%)	
Yes	38 (3.82%)	29 (3.55%)	9 (5.03%)	
Chest tightness, *n* (%)				0.198
No	601 (60.4%)	501 (61.4%)	100 (55.9%)	
Yes	394 (39.6%)	315 (38.6%)	79 (44.1%)	
Anhelation, *n* (%)				0.055
No	664 (66.7%)	556 (68.1%)	108 (60.3%)	
Yes	331 (33.3%)	260 (31.9%)	71 (39.7%)	
Hemoptysis, *n* (%)				0.548
No	991 (99.6%)	813 (99.6%)	178 (99.4%)	
Yes	4 (0.40%)	3 (0.37%)	1 (0.56%)	
Chest pain, *n* (%)				0.410
No	910 (91.5%)	743 (91.1%)	167 (93.3%)	
Yes	85 (8.54%)	73 (8.95%)	12 (6.70%)	
Syncope, *n* (%)				0.227
No	947 (95.2%)	773 (94.7%)	174 (97.2%)	
Yes	48 (4.82%)	43 (5.27%)	5 (2.79%)	
Cough, *n* (%)				0.163
No	744 (74.8%)	618 (75.7%)	126 (70.4%)	
Yes	251 (25.2%)	198 (24.3%)	53 (29.6%)	
Fever, *n* (%)				1.000
No	973 (97.8%)	798 (97.8%)	175 (97.8%)	
Yes	22 (2.21%)	18 (2.21%)	4 (2.23%)	
Lower limb edema, *n* (%)				0.003
No	842 (84.6%)	704 (86.3%)	138 (77.1%)	
Yes	153 (15.4%)	112 (13.7%)	41 (22.9%)	
COPD, *n* (%)				0.630
No	794 (79.8%)	654 (80.1%)	140 (78.2%)	
Yes	201 (20.2%)	162 (19.9%)	39 (21.8%)	
Hypertension, *n* (%)				0.706
No	382 (38.4%)	316 (38.7%)	66 (36.9%)	
Yes	613 (61.6%)	500 (61.3%)	113 (63.1%)	
Diabetes, *n* (%)				0.840
No	836 (84.0%)	687 (84.2%)	149 (83.2%)	
Yes	159 (16.0%)	129 (15.8%)	30 (16.8%)	
Coronary heart disease, *n* (%)				0.009
No	330 (33.2%)	286 (35.0%)	44 (24.6%)	
Yes	665 (66.8%)	530 (65.0%)	135 (75.4%)	
Hyperlipidemia, *n* (%)				1.000
No	969 (97.4%)	794 (97.3%)	175 (97.8%)	
Yes	26 (2.61%)	22 (2.70%)	4 (2.23%)	
Atrial fibrillation, *n* (%)				<0.001
No	763 (76.7%)	645 (79.0%)	118 (65.9%)	
Yes	232 (23.3%)	171 (21.0%)	61 (34.1%)	
Operation, *n* (%)				1.000
No	988 (99.3%)	810 (99.3%)	178 (99.4%)	
Yes	7 (0.70%)	6 (0.74%)	1 (0.56%)	
Tumor, *n* (%)				0.955
No	932 (93.7%)	765 (93.8%)	167 (93.3%)	
Yes	63 (6.33%)	51 (6.25%)	12 (6.70%)	
Smoking, *n* (%)				1.000
No	697 (70.1%)	572 (70.1%)	125 (69.8%)	
Yes	298 (29.9%)	244 (29.9%)	54 (30.2%)	
Drinking, *n* (%)				0.818
No	666 (66.9%)	548 (67.2%)	118 (65.9%)	
Yes	329 (33.1%)	268 (32.8%)	61 (34.1%)	
WBC (10^9^/L)	4.33 [3.93; 4.70]	4.33 [3.92; 4.71]	4.32 [3.97; 4.68]	0.799
RBC (10^12^/L)	7.28 [5.75; 9.64]	7.18 [5.61; 9.51]	7.61 [6.21; 10.7]	0.009
Mg (mmol/L)	0.90 [0.84; 0.95]	0.90 [0.84; 0.96]	0.88 [0.81; 0.93]	<0.001
HGB (g/L)	131 [119; 144]	132 [119; 145]	129 [119; 142]	0.295
Hct	0.40 [0.36; 0.43]	0.40 [0.36; 0.43]	0.40 [0.36; 0.43]	0.874
Neutrophil percent	0.73 [0.65; 0.82]	0.73 [0.64; 0.81]	0.76 [0.67; 0.84]	0.004
Neutrophil count (10^9^/L)	5.13 [3.75; 7.41]	5.03 [3.64; 7.16]	5.43 [4.26; 8.31]	0.003
Lymphocyte percent	0.23 [0.16; 0.31]	0.23 [0.17; 0.31]	0.21 [0.16; 0.29]	0.051
Lymphocyte count (10^9^/L)	1.46 [1.07; 1.96]	1.49 [1.09; 1.96]	1.38 [1.02; 1.89]	0.297
PLT (10^9^/L)	199 [161; 248]	198 [161; 246]	207 [165; 264]	0.193
PDW (%)	16.1 [15.4; 16.4]	16.1 [15.3; 16.4]	16.1 [15.6; 16.4]	0.306
RDW (%)	0.13 [0.13; 0.14]	0.13 [0.13; 0.14]	0.14 [0.13; 0.15]	<0.001
Fibrinogen (g/L)	3.53 [2.94; 4.40]	3.53 [2.97; 4.37]	3.67 [2.86; 4.52]	0.772
D-dimer (mg/L)	1.60 [0.87; 5.09]	1.35 [0.80; 3.93]	3.86 [1.63; 8.49]	<0.001
PT (s)	13.9 [13.2; 14.8]	13.8 [13.1; 14.6]	14.4 [13.6; 15.5]	<0.001
APTT (s)	37.6 [34.6; 41.2]	37.6 [34.6; 41.2]	37.7 [34.8; 41.4]	0.984
TT (s)	16.4 [15.8; 17.1]	16.4 [15.8; 17.1]	16.5 [15.8; 17.2]	0.413

Notes: COPD, chronic obstructive pulmonary disease; WBC, white blood cell count; RBC, red blood cell count; Mg, magnesium; HGB, hemoglobin; Hct, hematocrit; PLT, platelet count; PDW, platelet distribution width; RDW, red blood cell distribution width; PT, prothrombin time; APTT, activated partial prothrombin time; TT, thrombin time; PE, pulmonary embolism.

**Table 2:** Baseline characteristics of the enrolled patients in the training and validation cohorts

Variables	Total (*N* = 995)	Validation (*N* = 298, 30%)	Training (*N* = 697, 70%)	*p*
PE, *n* (%)				0.075
No	816 (82.0%)	234 (78.5%)	582 (83.5%)	
Yes	179 (18.0%)	64 (21.5%)	115 (16.5%)	
Sex, *n* (%)				0.895
Female	499 (50.2%)	148 (49.7%)	351 (50.4%)	
Male	496 (49.8%)	150 (50.3%)	346 (49.6%)	
Age (years)	76.0 [67.0; 82.0]	75.0 [66.2; 82.0]	76.0 [67.0; 82.0]	0.996
Breathing (breaths/min)	22.0 [20.0; 26.0]	22.0 [20.0; 26.0]	22.0 [20.0; 26.0]	0.400
Pulse (beats/min)	103 [89.0; 124]	103 [89.2; 121]	103 [89.0; 125]	0.704
Systolic pressure (mmHg)	101 [91.0; 113]	101 [91.0; 113]	100 [91.0; 113]	0.680
Diastolic pressure (mmHg)	54.0 [47.0; 63.0]	54.0 [48.2; 62.0]	54.0 [47.0; 64.0]	0.979
Headache, *n* (%)				0.547
No	968 (97.3%)	288 (96.6%)	680 (97.6%)	
Yes	27 (2.71%)	10 (3.36%)	17 (2.44%)	
Dizzy, *n* (%)				0.444
No	957 (96.2%)	284 (95.3%)	673 (96.6%)	
Yes	38 (3.82%)	14 (4.70%)	24 (3.44%)	
Chest tightness, *n* (%)				0.620
No	601 (60.4%)	184 (61.7%)	417 (59.8%)	
Yes	394 (39.6%)	114 (38.3%)	280 (40.2%)	
Anhelation, *n* (%)				0.496
No	664 (66.7%)	204 (68.5%)	460 (66.0%)	
Yes	331 (33.3%)	94 (31.5%)	237 (34.0%)	
Hemoptysis, *n* (%)				0.587
No	991 (99.6%)	296 (99.3%)	695 (99.7%)	
Yes	4 (0.40%)	2 (0.67%)	2 (0.29%)	
Chest pain, *n* (%)				0.796
No	910 (91.5%)	271 (90.9%)	639 (91.7%)	
Yes	85 (8.54%)	27 (9.06%)	58 (8.32%)	
Syncope, *n* (%)				0.968
No	947 (95.2%)	283 (95.0%)	664 (95.3%)	
Yes	48 (4.82%)	15 (5.03%)	33 (4.73%)	
Cough, *n* (%)				0.221
No	744 (74.8%)	231 (77.5%)	513 (73.6%)	
Yes	251 (25.2%)	67 (22.5%)	184 (26.4%)	
Fever, *n* (%)				0.325
No	973 (97.8%)	294 (98.7%)	679 (97.4%)	
Yes	22 (2.21%)	4 (1.34%)	18 (2.58%)	
Lower limb edema, *n* (%)				0.897
No	842 (84.6%)	251 (84.2%)	591 (84.8%)	
Yes	153 (15.4%)	47 (15.8%)	106 (15.2%)	
COPD, *n* (%)				0.248
No	794 (79.8%)	245 (82.2%)	549 (78.8%)	
Yes	201 (20.2%)	53 (17.8%)	148 (21.2%)	
Hypertension, *n* (%)				1.000
No	382 (38.4%)	114 (38.3%)	268 (38.5%)	
Yes	613 (61.6%)	184 (61.7%)	429 (61.5%)	
Diabetes, *n* (%)				0.868
No	836 (84.0%)	249 (83.6%)	587 (84.2%)	
Yes	159 (16.0%)	49 (16.4%)	110 (15.8%)	
Coronary heart disease, *n* (%)				0.221
No	330 (33.2%)	90 (30.2%)	240 (34.4%)	
Yes	665 (66.8%)	208 (69.8%)	457 (65.6%)	
Hyperlipidemia, *n* (%)				0.757
No	969 (97.4%)	289 (97.0%)	680 (97.6%)	
Yes	26 (2.61%)	9 (3.02%)	17 (2.44%)	
Atrial fibrillation, *n* (%)				0.327
No	763 (76.7%)	235 (78.9%)	528 (75.8%)	
Yes	232 (23.3%)	63 (21.1%)	169 (24.2%)	
Operation, *n* (%)				1.000
No	988 (99.3%)	296 (99.3%)	692 (99.3%)	
Yes	7 (0.70%)	2 (0.67%)	5 (0.72%)	
Tumor, *n* (%)				0.338
No	932 (93.7%)	283 (95.0%)	649 (93.1%)	
Yes	63 (6.33%)	15 (5.03%)	48 (6.89%)	
Smoking, *n* (%)				0.734
No	697 (70.1%)	206 (69.1%)	491 (70.4%)	
Yes	298 (29.9%)	92 (30.9%)	206 (29.6%)	
Drinking, *n* (%)				0.887
No	666 (66.9%)	198 (66.4%)	468 (67.1%)	
Yes	329 (33.1%)	100 (33.6%)	229 (32.9%)	
WBC (10^9^/L)	4.33 [3.93; 4.70]	4.34 [3.97; 4.68]	4.32 [3.92; 4.71]	0.883
RBC (10^12^/L)	7.28 [5.75; 9.64]	7.04 [5.74; 9.56]	7.41 [5.75; 9.64]	0.454
Mg (mmol/L)	0.90 [0.84; 0.95]	0.91 [0.83; 0.96]	0.90 [0.84; 0.95]	0.400
HGB (g/L)	131 [119; 144]	131 [120; 144]	131 [118; 145]	0.692
Hct	0.40 [0.36; 0.43]	0.39 [0.37;0.43]	0.40 [0.36; 0.43]	0.790
Neutrophil percent	0.73 [0.65; 0.82]	0.73 [0.64;0.83]	0.74 [0.65; 0.81]	0.542
Neutrophil count (10^9^/L)	5.13 [3.75; 7.41]	4.96 [3.64;7.34]	5.25 [3.78; 7.41]	0.250
Lymphocyte percent	0.23 [0.16; 0.31]	0.24 [0.16;0.32]	0.23 [0.16; 0.30]	0.249
Lymphocyte count (10^9^/L)	1.46 [1.07; 1.96]	1.46 [1.08;2.03]	1.46 [1.07; 1.94]	0.669
PLT (10^9^/L)	199 [161; 248]	197 [161;246]	200 [161; 248]	0.699
PDW (%)	16.1 [15.4; 16.4]	16.0 [14.7;16.4]	16.1 [15.6; 16.4]	0.018
RDW (%)	0.13 [0.13; 0.14]	0.13 [0.13;0.14]	0.13 [0.13; 0.14]	0.856
Fibrinogen (g/L)	3.53 [2.94; 4.40]	3.47 [2.89;4.32]	3.57 [2.97; 4.43]	0.236
D-dimer (mg/L)	1.60 [0.87; 5.09]	1.42 [0.83;4.89]	1.69 [0.87; 5.14]	0.257
PT (s)	13.9 [13.2; 14.8]	13.7 [13.1;14.7]	14.0 [13.2; 14.8]	0.092
APTT (s)	37.6 [34.6; 41.2]	37.4 [34.6;40.7]	37.7 [34.7; 41.4]	0.223
TT (s)	16.4 [15.8; 17.1]	16.5 [15.9;17.2]	16.4 [15.7; 17.0]	0.027

Notes: COPD, chronic obstructive pulmonary disease; WBC, white blood cell count; RBC, red blood cell count; Mg, magnesium; HGB, hemoglobin; Hct, hematocrit; PLT, platelet count; PDW, platelet distribution width; RDW, red blood cell distribution width; PT, prothrombin time; APTT, activated partial prothrombin time; TT, thrombin time.

### 3.2 Selected predictors and construction model

**Figure 2:**
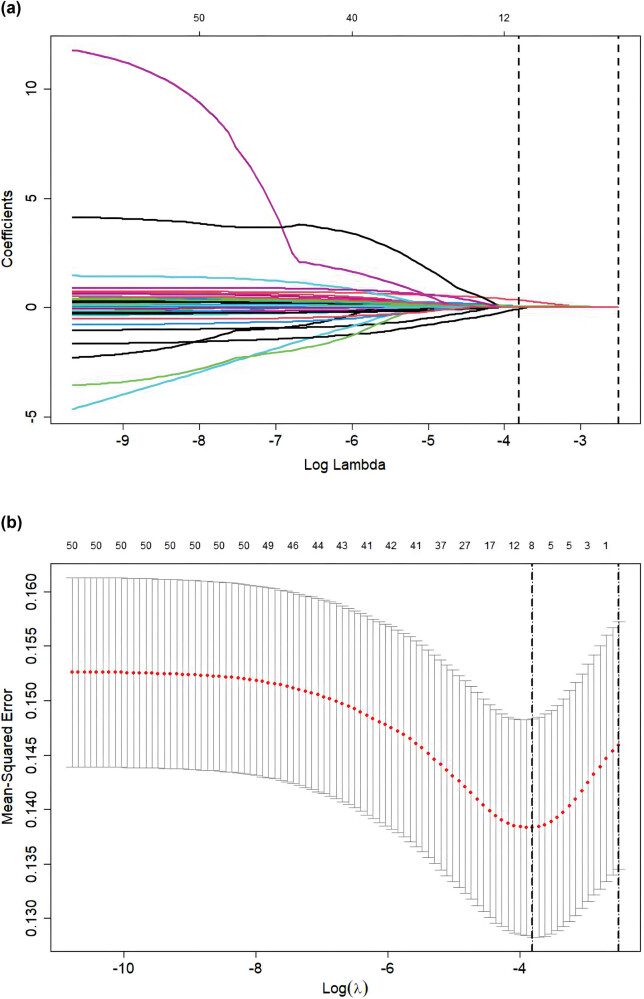
Tuning parameter selection using LASSO regression in the training cohort. (a) LASSO coefficient profiles of the clinical features. (b) Optimal penalization coefficient lambda was generated in LASSO through tenfold cross-validation. The lambda value of the minimum mean square error is shown in the figure.

After applying the LASSO regression analysis, we identified six of 45 variables that were potential predictive features (Figure 2a and b). The optimal predictors were age, pulse, systolic pressure, syncope, D-dimer, and coronary heart disease. These six potential predictive features were used to develop the final model based on the multivariable logistic regression analysis in the training cohort (Table 3). In the training cohort, our model had a sensitivity of 69.1%, a specificity of 63.4%, a positive predictive value of 28.8%, and a negative predictive value of 90.5%.

**Table 3:** Final model coefficients

Variables	*β*	SE	OR	95% CI	*p*
Age (years)	0.022	0.01048	1.022	1.002–1.044	0.034
Pulse (beats/min)	0.007	0.004	1.007	1.000–1.015	0.065
Systolic pressure (mmHg)	−0.014	0.00635	0.986	0.974–0.999	0.03
Syncope (yes or no)	−1.224	0.75105	0.294	0.067–1.282	0.103
CHD (yes or no)	0.709	0.2476	2.032	1.251–3.302	0.004
D-dimer (mg/L)	0.086	0.01947	1.089	1.049–1.132	<0.001

Notes: CI, confidence interval; OR, odds ratio; SE, standard error; CHD, coronary heart disease.

### 3.3 Model visualization

**Figure 3:**
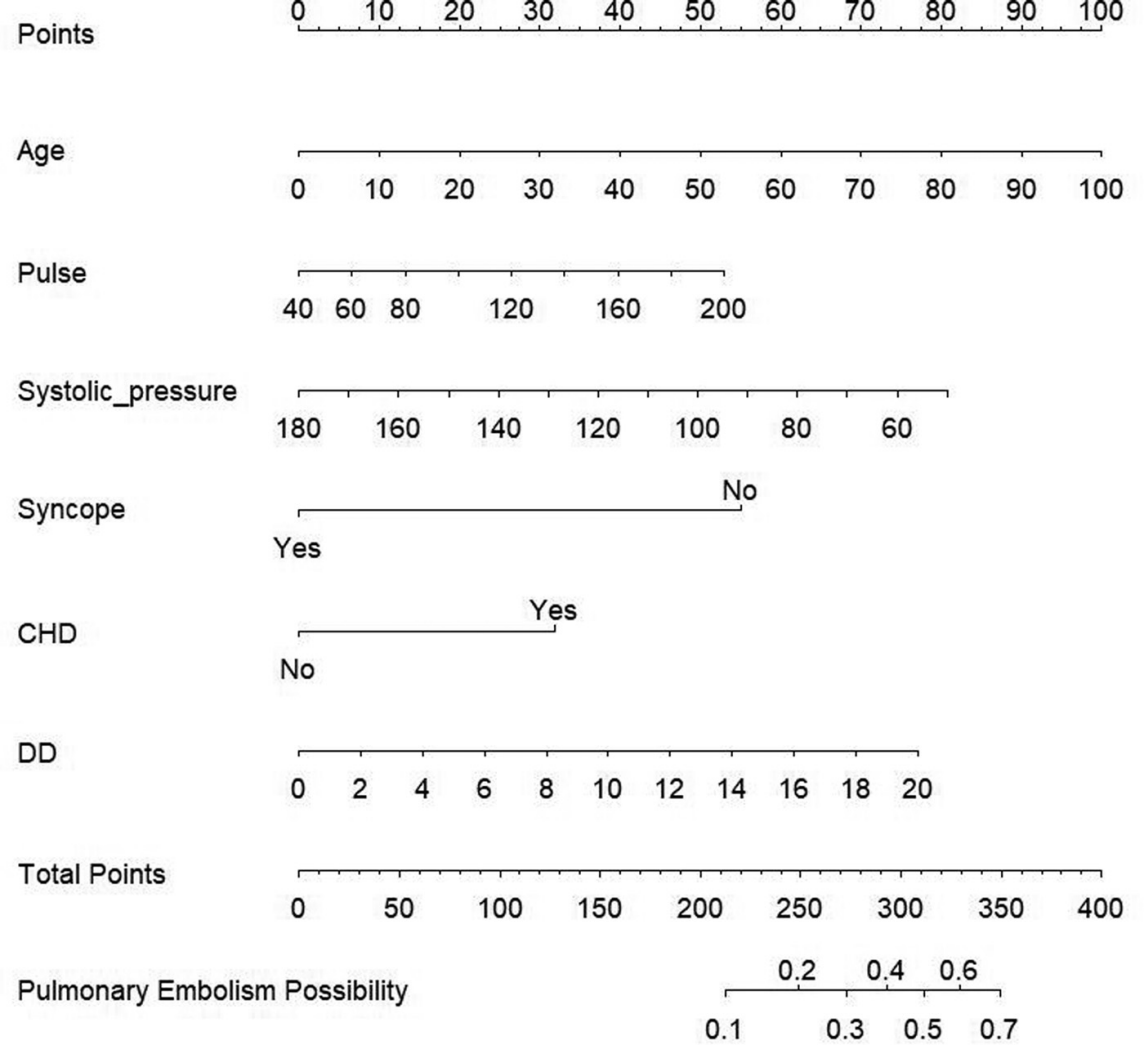
Nomogram based on the combination of the six indicators was developed using logistic regression analysis. If a patient has a total score of 250, then the probability of developing PE is 0.21. DD, D-dimer; CHD, coronary heart disease.

Multivariate analysis revealed that age (OR = 1.022, 95% CI, 1.002–1.044), pulse (OR = 1.007, 95% CI, 1.000–1.015), systolic pressure (OR = 0.986, 95% CI, 0.974–0.999), D-dimer (OR = 2.032, 95% CI, 1.251–3.302), and coronary heart disease (OR = 1.089, 95% CI, 1.049–1.132) were independent predictors for PE (Table 3). The nomogram (Figure 3) shows the predictive model for PE based on the six selected variables: age, pulse, systolic pressure, syncope, D-dimer, and coronary heart disease. To use the nomogram, each variable was assigned a score based on its value, and the scores were summed to obtain a total score. A vertical line was then drawn from the total score axis to the probability axis to obtain the estimated probability of PE. For example, if a patient is 80 years old, has a pulse rate of 160 beats per minute, systolic pressure of 82 mmHg, no history of syncope, a D-dimer level of 4.3 mg/L, and no history of coronary heart disease, then the total score would be 250. The vertical line from the total score of 250 intersects the probability axis at approximately 0.21, indicating a 21% estimated probability of PE.

### 3.4 Model validation and evaluation

**Figure 4:**
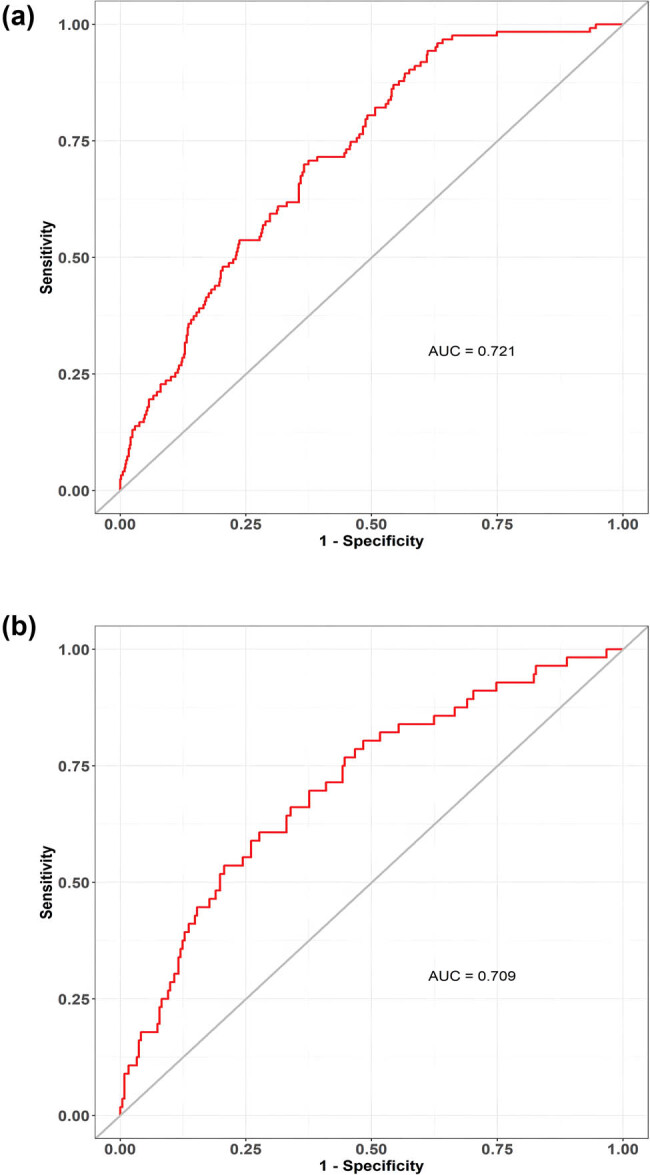
ROC curves of the model to distinguish PE from non-PE in the training (a) and validation (b) cohorts.

**Figure 5:**
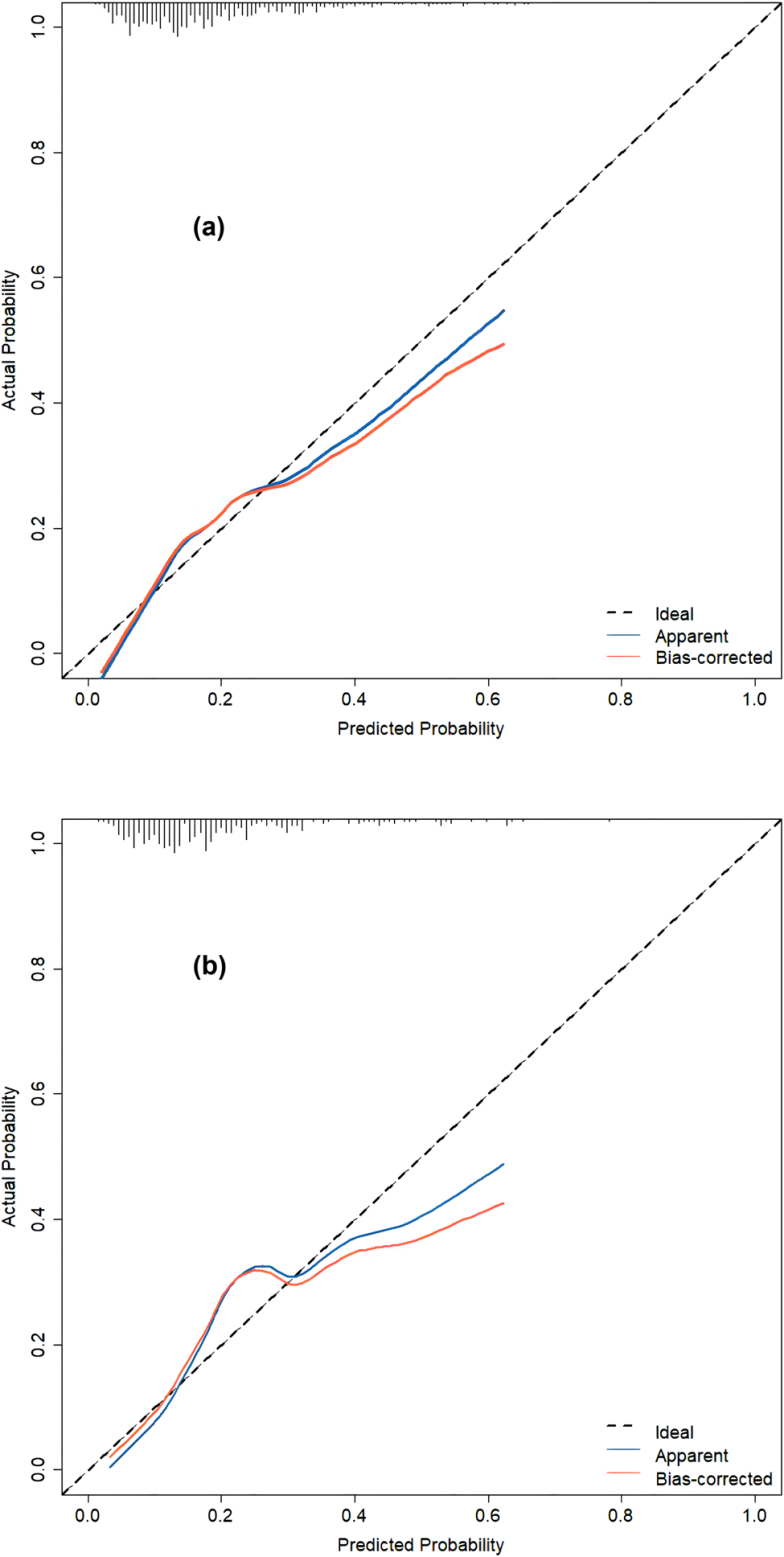
Calibration curves of the model in the training (a) and validation (b) cohorts. A perfect accurate predictive model will generate a plot where the probability of the actual observed and prediction completely fall along the ideal line (dashed line). The apparent calibration curve (blue line) represents the calibration of the model, while the bias-corrected curve (red line) is the calibration result after correcting the optimism with fivefold cross-validation.

**Figure 6:**
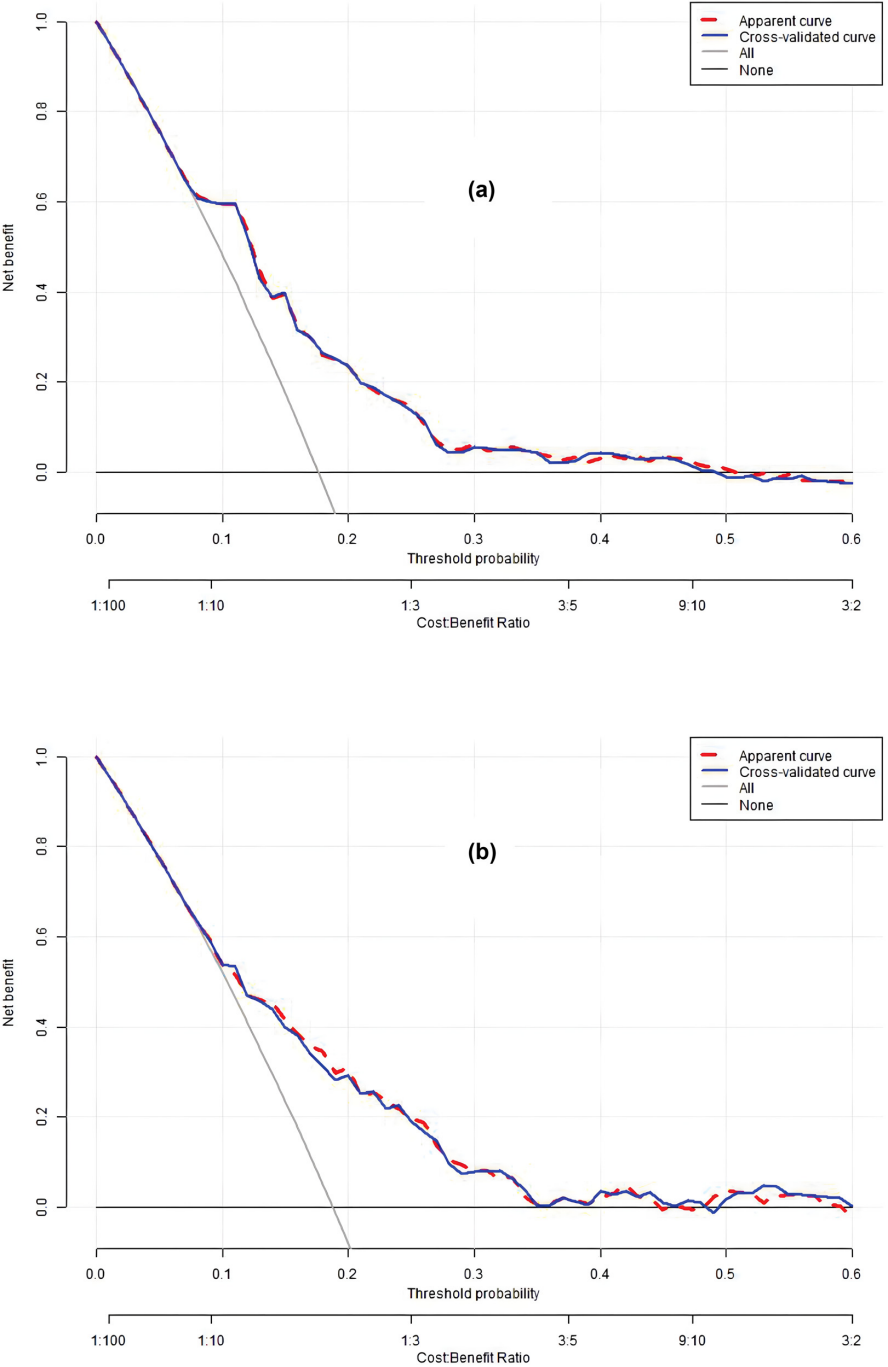
Decision curve of the model in the training (a) and validation (b) cohorts. The red line is a nomogram net clinical benefit of PE, while the cross-validated curve (blue line) is a net clinical benefit of PE after correcting the optimism with fivefold cross-validation. The solid gray line indicates that all patients had PE, while the fine solid black line indicates that no patient had PE. This DCA could provide a larger net benefit, in the range of 8–50%. If the risk threshold is less than 50%, then the nomogram model will obtain more benefit than all treatment (assuming all patients were PE) or no treatment (assuming all patients were non-PE).

**Figure 7:**
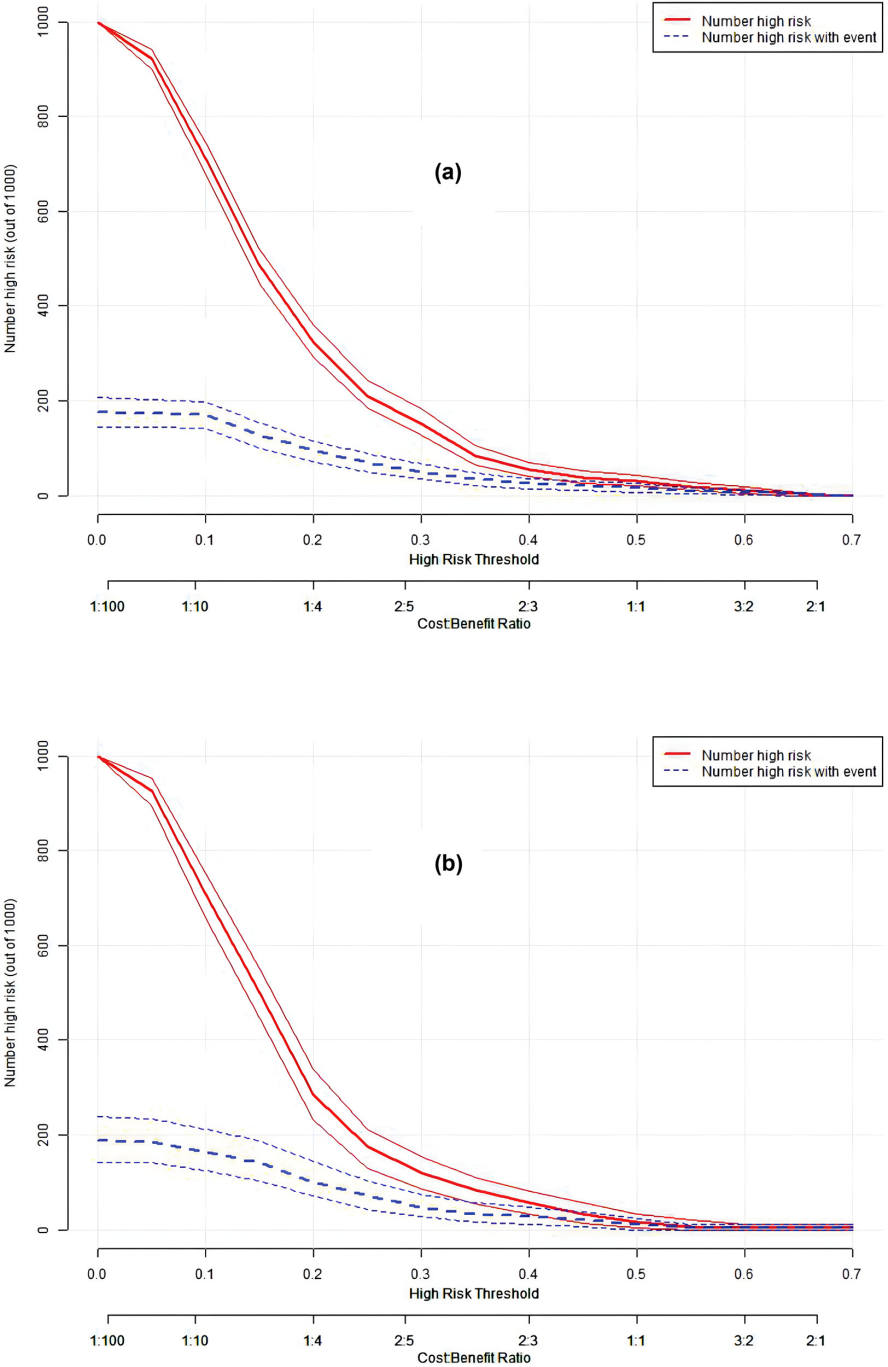
Clinical impact curve of the model in the training (a) and validation (b) cohorts. The red line indicates the number of subjects who are judged as being at high risk by the model under different probability thresholds. The blue line indicates the number of subjects who are judged by the model to be at high risk and who actually have an outcome event under different probability thresholds.

**Figure 8:**
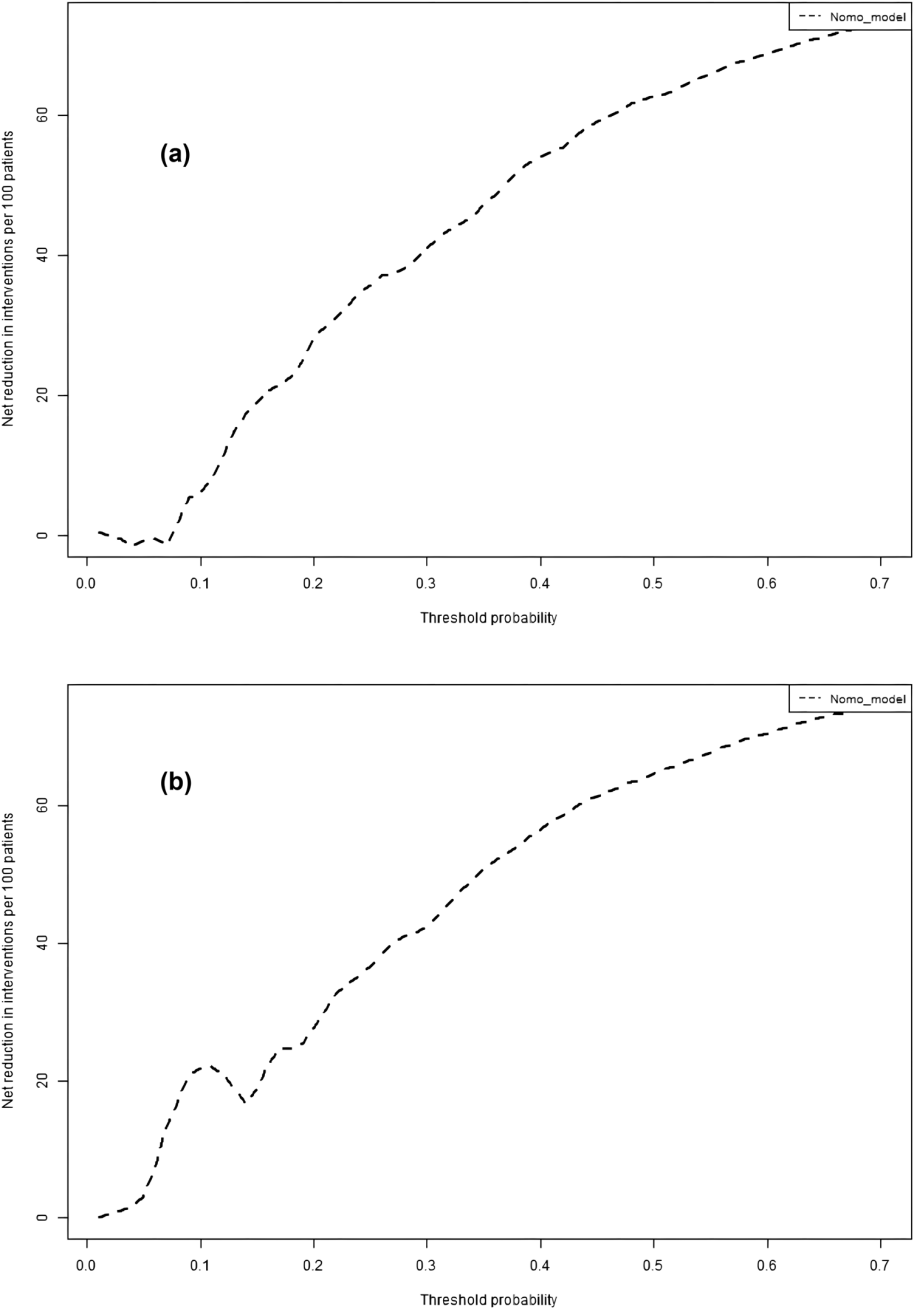
Net reduction curve of the model in the training (a) and validation (b) cohorts. Using predictive models can reduce the number of interventions by 40% at a risk threshold of 30%.

The discriminatory ability of the numerical model, as measured by the AUC, was 0.721 (95% CI, 0.676–0.766) in the training cohort and 0.709 (95% CI, 0.633–0.784) in the validation cohort, indicating that the model can effectively differentiate between PE and non-PE cases (as illustrated in Figure 4a and b). The calibration plots, shown in Figure 5a and b, reveal good consistency between predicted probabilities and actual outcomes for both the training and validation cohorts, as evidenced by the proximity of the apparent calibration curve to the ideal line. The DCA curves, presented in Figure 6a and b, demonstrate that the numerical model had a favorable net clinical benefit, with screening strategies based on our nomogram PE risk estimates yielding greater net benefit than both screen-none and screen-all strategies within the threshold probability range of 0.08–0.50. Furthermore, the clinical impact curve and net reduction curve depicted in Figures 7 and 8, respectively, indicate that our nomogram has a significant net clinical benefit.

## 4 Discussion

In this study, we developed a novel predictive model for the probability of PE in patients with cardiovascular diseases. Our model utilized six variables representing high-risk disease, namely age, pulse, systolic pressure, syncope, D-dimer, and coronary heart disease, all of which are easily obtainable clinical features and biomarkers during routine health assessments. Our findings showed that the model exhibited good discrimination with an area under the ROC curve of 0.721 (95% CI, 0.676–0.766), indicating its ability to distinguish between patients with and without PE. Furthermore, the calibration plots demonstrated that the model had a good consistency between predicted and observed probabilities in both training and validation cohorts. Additionally, the DCA suggested that our model had a favorable net clinical benefit within the threshold probability range of 0.08–0.50.

Currently, there are no predictive models available for predicting the risk of PE specifically in patients with cardiovascular disease. However, previous studies have developed predictive models for venous thromboembolism in other patient populations. For instance, Zhou et al. [22] developed a predictive model for PE in patients with cough or chest pain based on laboratory variables, which had an AUC of 0.692. Li et al. [23] developed a clinical predictive model for lower extremity deep venous thrombosis in patients admitted to the neurointensive care unit, which had an AUC of 0.817. Zhang et al. developed a predictive model for postoperative venous thromboembolism [24].

Our study has several strengths compared to previous studies. First, we utilized the LASSO regression method to select the optimal predictive features, which improved the accuracy and robustness of the predictive models. Second, our model used only six readily available high-risk variables, which makes it simpler and more efficient to use in clinical practice. Finally, our model specifically focuses on the prediction of PE in patients with cardiovascular disease, which makes it highly relevant for clinicians dealing with this population.

D-dimer is an indicator reflecting fibrinolytic function and can be used to diagnose thrombotic diseases. According to our study, D-dimer (OR = 2.032, 95% CI, 1.251–3.302) was identified as an independent predictor for increased risk of PE. This finding is consistent with those of previous research [25] that has linked high D-dimer levels with increased risk of developing PE. Although D-dimer is currently the only biomarker used in routine clinical practice to predict PE, its specificity is limited, leading to high rates of false-positive results. Elderly patients have increased hospitalization rates and the highest inpatient mortality due to PE, as demonstrated in a large-sample study conducted from 2000 to 2015 [26]. Another retrospective study indicated that age is associated with the severity of submassive PE stadium [27], and our model also found age (OR = 1.022, 95% CI, 1.002–1.044) to be a high-risk factor for PE, which is consistent with the findings of previous research. Most of the factors in our model were positively associated with the risk of PE, except for systolic blood pressure, which was negatively associated. Low systolic pressure has been linked to an increased risk of PE-related mortality, as shown in a previous study [28]. The relationship between lower systolic blood pressure and higher PE occurrence is primarily due to the pathophysiology of PE itself. When a blood clot (or embolus) travels through the bloodstream and lodges in the pulmonary arteries, it prevents effective oxygen exchange in the lungs. This can lead to acute right heart failure because the right side of the heart has to pump harder against the increased resistance in these blocked arteries. Our data also revealed that pulse rate was included in the model to predict PE. Consistent with our findings, a previous study [29] identified pulse rate as a good predictor of PE. This happens because when a blood clot obstructs the pulmonary arteries, the right ventricle of the heart has to work harder to pump blood through these vessels. This increased workload can result in a faster heart rate. Coronary heart disease [30] has also been identified as a factor that affects the risk of PE, which is similar to the indicators present in our model. Our study obtained an interesting result that patients with syncope are less likely to develop PE. Syncope is a common clinical symptom of PE [31], but our findings differ from that generally observed. We analyzed the clinical information of 48 patients with syncope and found that the possible explanation for this interesting result is that these patients mainly had vasovagal syncope or orthostatic hypotension syncope. They were sent for CTPA only for exclusion and did not have a high suspicion of PE. Additionally, patients with PE who experienced syncope were sent to the respiratory department for treatment.

The development of an accurate model for predicting the probability of PE in patients with cardiovascular disease has significant implications in clinical practice. This model can aid healthcare professionals in making timely and personalized diagnoses, leading to the formulation of effective and personalized treatment plans. By identifying high-risk variables such as age, pulse, systolic pressure, syncope, D-dimer, and coronary heart disease, this model can assist in identifying patients who require further diagnostic workup or more aggressive treatment, while reducing the need for unnecessary CTPA screening. Furthermore, the use of a nomogram to visualize the model’s output makes it easier for healthcare professionals to interpret the results and communicate them to patients. The high net clinical benefit demonstrated by the clinical decision curve, clinical impact curve, and net reduction curve analyses suggests that this model has the potential to improve patient outcomes and reduce healthcare costs [32].

However, there are some limitations to our study. For example, the sample size of this study was smaller than that in some previous studies, which may limit the generalizability of our findings. Additionally, because our model was developed using retrospective data, there may be a risk of selection bias and confounding. Finally, external validation in other clinical settings is required to assess the generalizability and reliability of our model.

In conclusion, the novel model developed in this study has the potential to become a valuable clinical tool for predicting the probability of PE in patients with cardiovascular diseases, leading to more accurate diagnoses and personalized treatment plans. However, it is important to note that this study is retrospective, and further prospective studies are required to validate the accuracy and clinical usefulness of this model.

## Abbreviations

CHD: coronary heart disease
CI: confidence interval
CTPA: computed tomography pulmonary angiography
DCA: decision curve analysis
LASSO: least absolute shrinkage and selection operator
OR: odds ratios
PE: pulmonary embolism
ROC: receiver operating characteristic
